# Effect of Beta Radiation on the Quality of the Bonded Joint for Difficult to Bond Polyolefins

**DOI:** 10.3390/polym11111863

**Published:** 2019-11-12

**Authors:** David Manas, Martin Bednarik, Ales Mizera, Miroslav Manas, Martin Ovsik, Pavel Stoklasek

**Affiliations:** 1 Faculty of Technology, Tomas Bata University in Zlin, Vavreckova 275, 760 01 Zlin, Czech Republic; manas@utb.cz (D.M.); ovsik@utb.cz (M.O.); 2 CEBIA-Tech, Faculty of Applied Informatics, Tomas Bata University in Zlin, Nad Stranemi 4511, 760 05 Zlin, Czech Republic; mizera@utb.cz (A.M.); manas@fai.utb.cz (M.M.); pstoklasek@utb.cz (P.S.)

**Keywords:** adhesion, radiation crosslinking, beta radiation, wetting contact angle, free surface energy, oxidation, bonded joints, polyethylene, polypropylene

## Abstract

Bonding is increasingly being used, and it is an ever-evolving method for creating unbreakable bonds. The strength of adhesive bonds determines, to a significant extent, the possible applications of this technology and is influenced by many factors. In addition to the type of adhesive used, the characteristics of the surface layers play a significant role; therefore, significant attention is paid to their adjustment and modification. Radiation crosslinking is one of the most important methods for modifying polymer properties. Currently, the most frequently used type of radiation for polymer crosslinking is beta minus (β^−^) radiation, which affects not only mechanical but also surface properties, chemical and temperature resistance, and surface layer characteristics of polymers. This study investigated the effect of β^−^ radiation on the surface layer properties of low-density polyethylene (LDPE), high-density polyethylene (HDPE), and polypropylene (PP) and the effects of surface-layer modification on the ultimate tensile strength of bonded joints. Based on the results, we concluded that β^−^ radiation significantly changes the properties of the tested surface layers, increases the surface energy, and improves the adhesiveness of bonds. Consequently, the final strength of the LDPE, HDPE, and PP bonds increases significantly.

## 1. Introduction

Nowadays, polymer materials are used for a wide range of industrial applications and a wide variety of purposes, including packaging materials, household products, and sports products, as well as for very demanding applications in healthcare, the automotive, aerospace, electrotechnics, and electronics industries. Individual parts often need to be joined together into larger units, using dismountable and non-dismountable bonds. In particular, welding and bonding technologies are used to create non-dismountable bonds. The ability to form sufficiently rigid bonds depends on the adhesive used and surface-layer properties of the bonded materials, and, in particular, for polymeric materials, it is necessary to modify the surfaces of the adhered components. Modifying the surface layers allows the creation of controlled interfaces, which could be used to achieve desired properties, such as compatibility and adhesiveness [1,2].

From the adhesion and associated adhesiveness viewpoint, the fundamental difference between metals and plastics is their surface energy. The surface energies of polymers are lower than those of metals, and thus, polymers tend to form very poor-quality adhesive bonds. Hence, polymers often require suitable coatings [3,4]. A variety of methods are used to treat polymer-material surfaces, to improve their adhesion properties, including primarily corona discharge, flame exposure, and plasma and chemical etching. Nevertheless, not all methods present the same commercial uses, since their range of use is limited. For example, plasma treatment is limited to small parts and components, while plasma treatment and corona discharge are more effective for treating continuous strips and thin layers [2,4].

Lapcikova et al. [5] showed the positive effect of plasma surface treatment on polyethylene (PE), polypropylene (PP), and polycarbonate materials. This surface modification treatment led to significant reductions in wetting contact angles, which ultimately led to the significant increase in the bond strength of the bonded joints. The positive effect of these surface-modification methods on adhesive properties was described in many papers [6,7,8,9,10,11], and it is clear that these methods increased surface energies and improved surface-layer wettability, thus resulting in improved adhesive qualities, increased strength, and improved glued-bond quality. The influence of surface treatments of polymeric plasma filaments at atmospheric pressure on adhesion in matrices of rubber mixtures and other materials was addressed in several papers [12,13,14,15,16]. The influence of plasma on the surface layers of polyethylene terephthalate, at atmospheric pressures in nitrogen/butadiene mixture, was described by many scholars [17,18,19,20].

In addition to the abovementioned methods, other methods which are not primarily designed to improve the surface and adhesion properties but to improve the mechanical properties, as well as the temperature and chemical resistance of materials, can be used. These include, in particular, radiation crosslinking of polymers. The modification of polymer properties by using radiation is an ongoing research area which is gaining increasing interest from the industry [21,22,23,24,25,26,27]. Furthermore, the surface properties of polymers are significantly affected by radiation.

Ionizing radiation are often used to modify the polymer properties. Commonly, beta minus (β^−^) radiation is widely used for this purpose. The emitted electrons are accelerated in an electrostatic field between a cathode and an anode (see Section 2.3.). In general, the penetration depth depends on the density and the atomic number of the irradiated material, and the geometry could also have some interests affected, as reported in [21,22,23,24,25]. Most previous studies [28,29,30,31,32] that examined the effect of irradiation on the resulting properties of materials primarily focused on describing the structural changes, mechanical properties, and chemical- and temperature-resistance dependence on radiation doses. Gheysari et al. [23] referred the positive effect of high-energy β^−^ radiation on the mechanical and thermal properties of low-density and high-density polyethylene (LDPE and HDPE, respectively), which were modified by using radiation doses ranging from 50 to 250 kGy.

However, the effect of this type of modification on the properties of the surface layer, in particular on the surface energy, wettability, adhesive properties, and adhesiveness of polymers, has not been investigated yet. For industrial applications, it would be very beneficial if one type of modification could enhance the properties of surface layers, in addition to improving the mechanical properties, as well as the thermal and chemical resistance, of polymers.

This study focused on the influence of irradiation on the properties of surface layers, such as the wetting contact angle, free surface energy, and adhesiveness, of selected types of polyolefin polymeric materials, and built on the work of Bednarik et al. [33,34,35,36,37]. The main goal of this study was, therefore, to provide a comprehensive overview of the influence of irradiation on the surface properties of selected nonpolar polymeric materials, as well as on the relationship between the ultimate tensile strength of bonded joints and absorbed dose of radiation when using commercially available types of adhesives.

## 2. Materials and Methods

### 2.1. Polymers

Three polyolefin representatives which presented difficult adhesiveness were selected as test materials: LDPE, HDPE, and PP (see Table 1). These materials exhibit nonpolar characteristics and poor adhesion properties, which result in difficult adhesion without prior surface treatment.

### 2.2. Adhesives

Three groups of commercially available adhesives, cyanoacrylate-, methyl-acrylate-, and epoxide-based, were used to create adhesive bonds (see Table 2).

### 2.3. Test Specimen Preparation

**Figure 1:**
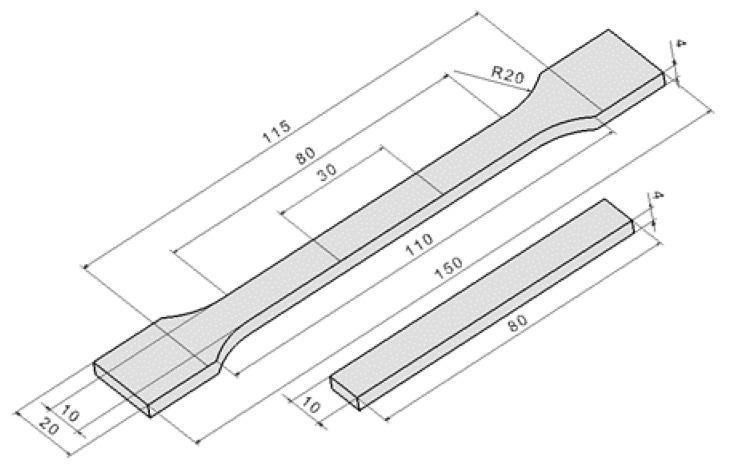
Test specimens.

Test specimens were used to obtain bonded joints for tensile tests (see Figure 1). The specimens were produced by using injection molding utilizing test polymers granules. The processing parameters are summarized in Table 3. The injected parts were irradiated at BGS Beta-Gamma-Service, Germany. Accelerated electron beams featuring various doses were used for all tests. The maximum dose of 198 kGy was used for HDPE and LDPE, while the maximum dose of 99 kGy was used for PP. The source of accelerated electrons was a Rhodotron high-voltage accelerator, which presented the maximum energy of 10 MeV. Crosslinking of HDPE and LDPE was performed without using a crosslinking agent, while PP contained 4% triallylisocyanurate as its crosslinking agent. The adequate radiation dose was determined by using a Nylon FTN 60-00 dosimeter. The analysis of absorbed radiation dose by dosimeter was performed by using a Genesys 5 spectrophotometer, in accordance with the ASTM 51261 standard [38], and the gel content was measured according to the CSN EN ISO 10147 standard [39], which involved solvent extraction, using xylene.

**Figure 2:**
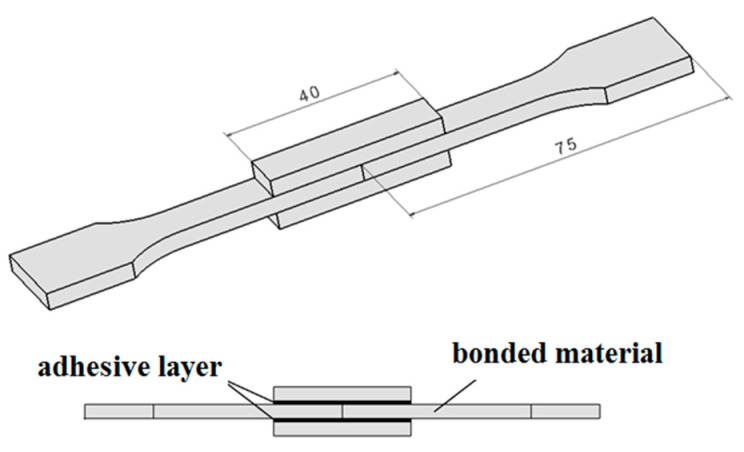
Bonded joint.

**Figure 3:**
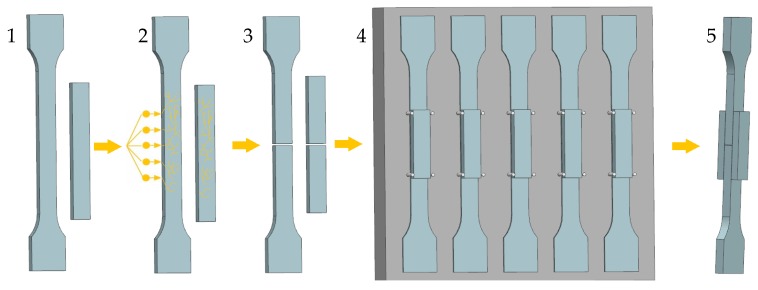
Test specimen preparation: (**1**) injection moulding, (**2**) irradiation, (**3**) cutting, and (**4**,**5**) bonded joint formation.

Bonded joints were formed by overlapping non-irradiated and irradiated specimens (see Figure 2). The constant thickness of the adhesive layers was ensured by placing spacers between the glued surfaces. The preparation procedure of the test specimens is presented in Figure 3.

### 2.4. Gel Content Measurements

The gel content was measured in accordance with the CSN EN ISO 10147 standard [39]. The examined sample was placed in xylene, for 8 h, at the boiling point of the solvent (142 °C), and then dried in a vacuum oven for 3 h, at 90 °C. The degree of crosslinking was expressed as the percentage of undissolved material.

### 2.5. Wetting Contact Angle Measurements

**Figure 4:**
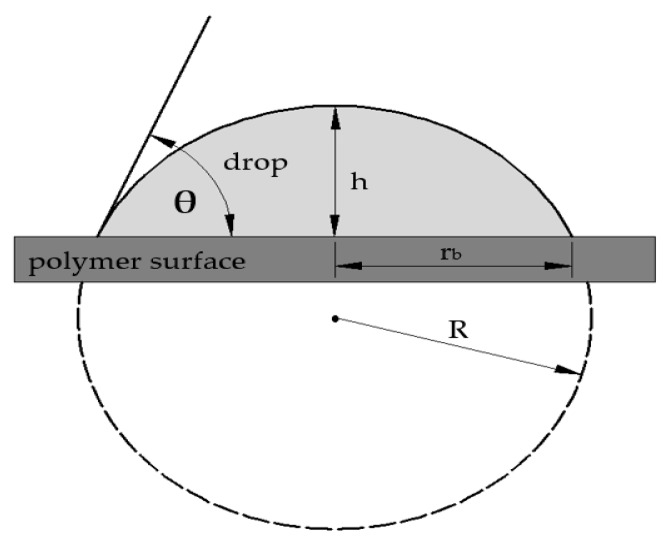
Droplet profile analysis: θ—wetting contact angle, *h*—droplet height, *r*_b_—droplet radius at contact point, and R—entire droplet radius.

The wetting contact angle, which characterizes the wetting ability of surfaces, was measured using sexagesimal system and the Sessile drop method, employing a See System instrument (Advex Instruments, Brno, Czech Republic). The measurements were carried out according to the CSN EN 15802 standard [40], using three reference liquids (distilled water, glycerin, and ethylene glycol), which featured different surface tensions. The measuring liquid drops were applied onto the surfaces of the polymers, using a micropipette, and the volume of each individual drop was 4 μL. The analysis of the droplet profile used to calculate the wetting contact angles is illustrated in Figure 4.

The drop height (*h*), its radius at the contact point (*r*_b_), and wetting contact angle (θ) were determined by using the following equations [41]:

(1) h=R(1−cosθ),

(2) $$r_{b}=Rsin\theta ,$$

and

(3) $$\frac{h}{r_{b}}=\frac{1-cos\theta }{sin\theta }=tan\left(\frac{\theta }{2}\right).$$

### 2.6. Determination of Surface Energies

The Owens–Wendt–Rabel–Kaelble (OWRK) regression method [42,43,44], which used the measured wetting contact angles, was employed to determine the surface energies of the samples. According to this method, the surface tension is given by the sum of the dispersed and polar components, γ_d_ and γ_p_, respectively. The following equations can be used for the surface tension of liquid and solid materials (γ_l_ and γ_s_, respectively) [42,43,44]:

(4) $$\gamma_{l}=\gamma_{l}^{d}+\gamma_{l}^{p},$$

(5) $$\gamma_{s}=\gamma_{s}^{d}+\gamma_{s}^{p},$$

(6) $$\gamma_{l}\left(1+cos\theta \right)=2\sqrt{\gamma_{s}^{d}\gamma_{l}^{d}}+2\sqrt{\gamma_{s}^{p}\gamma_{l}^{p}},$$

and

(7) $$\left(\gamma_{l}^{d}+\gamma_{l}^{p}\right)\left(1+cos\theta \right)=2\sqrt{\gamma_{s}^{d}\gamma_{l}^{d}}+2\sqrt{\gamma_{s}^{p}\gamma_{l}^{p}}.$$

**Figure 5:**
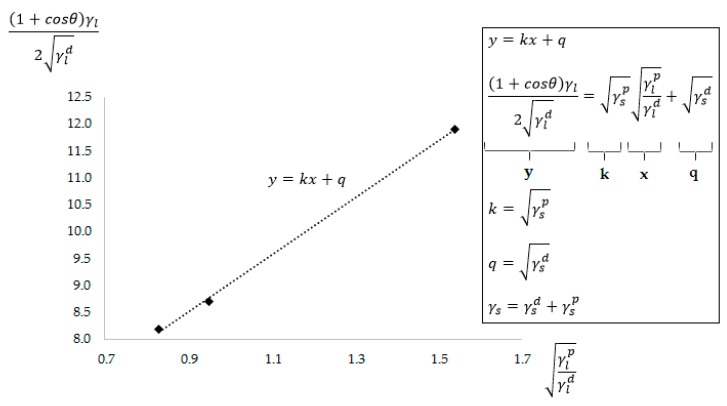
Determination of free surface energy using linear regression.

The above equations were further modified to achieve a linear equation. The resulting surface energy values were subsequently determined by using linear regression (see Equation (8) and Figure 5), which required the use of three types of reference liquids for calculations. The possible errors that could have occurred when using only two fluids, which could have been caused by their inappropriate combination, have been, therefore, eliminated by using this method [2,4,42,43,44,45].

(8) $$\frac{\left(1+cos\theta \right)\gamma_{l}}{2\sqrt{\gamma_{l}^{d}}}=\sqrt{\gamma_{s}^{p}}\sqrt{\frac{\gamma_{l}^{p}}{\gamma_{l}^{d}}}+\sqrt{\gamma_{s}^{d}}$$

### 2.7. Infrared Spectroscopy

Infrared spectroscopy was used to determine the relative numbers of hydroxyl and carbonyl groups on the surfaces of the analyzed polymers. The infrared spectra were measured using the attenuated total reflection (ATR) technique. A Nicolet 6700 FTIR spectrometer was purged using dry air and was used to obtain the infrared spectra, which were measured at the resolution of 2 cm^−1^, using 64 spectra accumulations. The background was a pure ATR diamond crystal. Spectra were processed by using the OMNIC software 8.2, while ATR corrections were used to modify them. The average of six measured spectra was used to determine the bond areas. Prior to averaging, the spectra were automatically adjusted to baseline and normalized.

### 2.8. Measurement of Load-Bearing Capacity of Bonded Joints

To assess the load-bearing capacity of the bonded joints, the maximum load force, which was defined by using the adhered joint, was measured. Tensile tests were carried out by using a Zwick 1456 tensile machine at the crossbar speed of 50 mm/min. Measurements were performed at room temperature (23 °C), and the data obtained were then evaluated by using the Test Expert software.

## 3. Results

This study analyzed the influence of β^−^ radiation on the surface properties and resulting resistance of bonded joints of selected types of polymeric materials. The HDPE, LDPE, and PP polyolefin materials were selected for their bonding difficulties. The influence of β^−^ radiation dose on the load-bearing capacity of the bonded joints was investigated. The selected polymers were modified by using radiation at the doses of 33, 66, 99, 132, 165, and 198 kGy for the HDPE and LDPE samples, and 33, 66, and 99 kGy for the PP samples. The changes in properties of the surface layers after irradiation and the effect of the modification on the load-bearing capacity of the bonded joints were studied.

### 3.1. Gel Content

**Figure 6:**
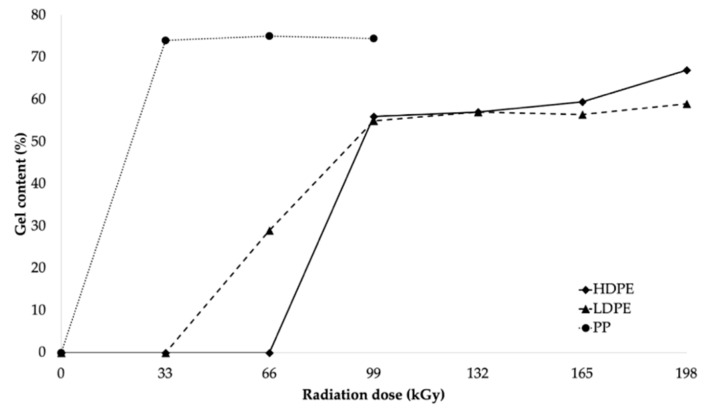
Gel content dependence on radiation dose.

The dependence of the gel content on the absorbed radiation dose for all tested materials is illustrated by the trend curves in Figure 6. The gel content depended on the radiation dose. Crosslinking predominantly occurs in amorphous areas [46]. The HDPE and LDPE test specimens that were irradiated by using the dose of 198 kGy exhibited the highest gel content. 

The gel contents of the HDPE and LDPE specimens irradiated using the dose of 198 kGy were 67% and 59%, respectively. No measurable gel content was identified for the HDPE and LDPE samples until the radiation dose was 66 and 33 kGy, respectively. This finding, however, seems to contrast with the results reported by Gheysari et al. [23], who noted changes in the mechanical properties of polymers at the lowest radiation doses, and, thus, the gel content was expected to partly correspond to those changes. This could be explained, to some extent, by the fact that the resulting micro-gels were not captured, and, therefore, not included in the total gel content.

The results for PP (see Figure 6) indicate that a significant increase in gel content could already be observed at the lowest radiation dose (33 kGy). At higher radiation doses (66 and 99 kGy), the changes in gel-phase content of PP were minimal (the gel content of the samples irradiated by using the doses of 66 and 99 kGy only increased by 3% compared to the dose irradiated by using the dose of 33 kGy). The maximum gel content of PP was 75% at the dose of 66 kGy. The changes listed above appear primarily in changes in the mechanical properties and in the increases in temperature and chemical resistance [23,46].

### 3.2. Surface-Layer Properties

**Figure 7:**
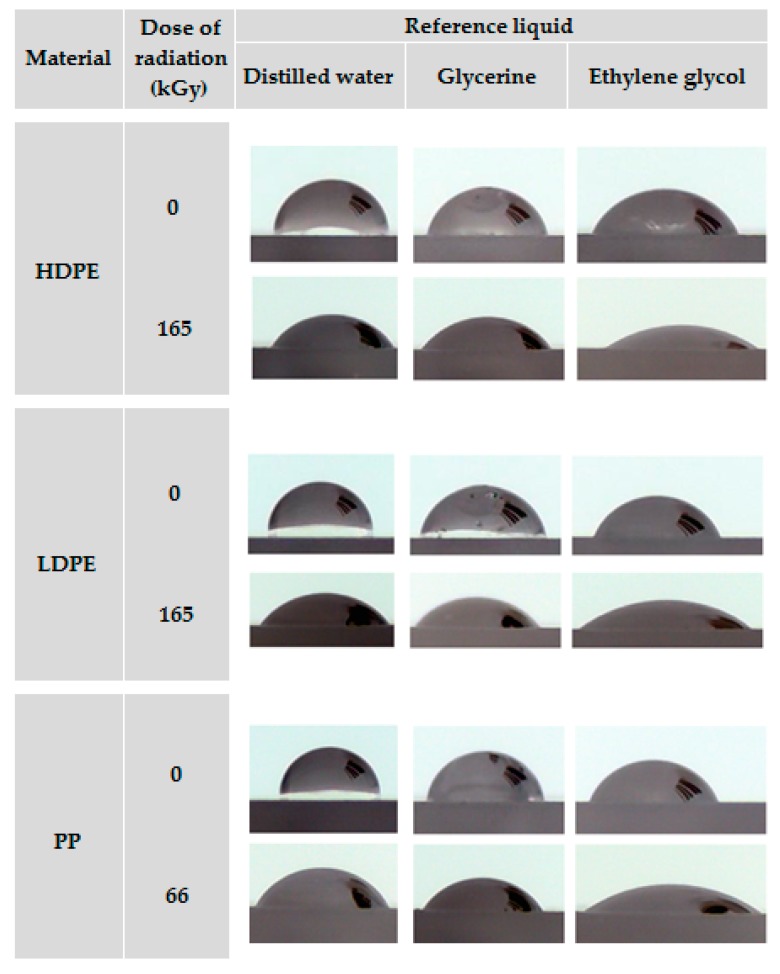
Liquid droplets on surfaces of test materials.

The effect of β^−^ radiation on surface properties was assessed by using the wetting contact angles, free surface energy, and relative numbers of carbonyl and hydroxyl groups. The changes in wetting contact angles of HDPE, LDPE, and PP are presented in Table 4 and Figure 7.

The measurement results indicated that β^−^ radiation significantly improved the wettability of all tested materials, which was reflected by the decrease in the wetting contact angles for all the reference liquids used. The most appropriate radiation dose for HDPE and LDPE was 165 kGy, which was associated with the most significant decrease in wetting contact angle of approximately 31% to 53% compared with the unmodified material, depending on the type of liquid used. Further increasing the radiation dose no longer caused the wetting contact angles to decrease.

A significant drop in the wetting contact angle of PP was recorded at the radiation dose of 33 kGy. However, the lowest wetting contact angles were achieved at the irradiation dose of 66 kGy: 59.5°, 55.9°, and 36.6° for distilled water, glycerin, and ethylene glycol, respectively. Depending on the type of liquid used, these angles were approximately 32% to 43% smaller than those of non-irradiated PP.

**Figure 8:**
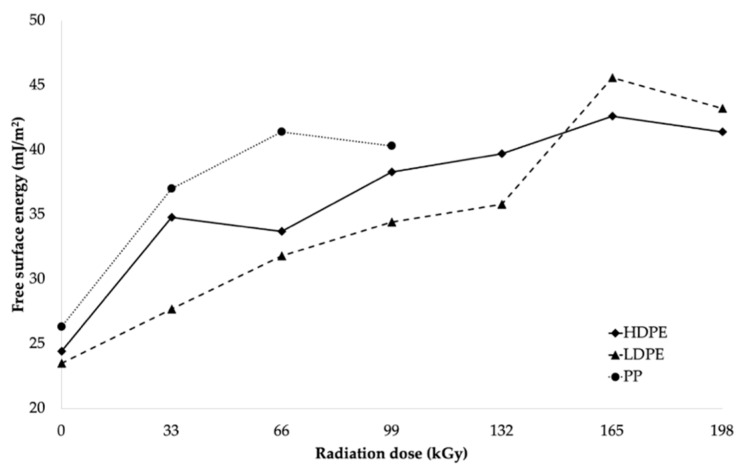
Free surface energy dependence on radiation dose.

The decrease in wetting contact angles was also closely related to the change in free surface energy of the tested materials, as illustrated in Table 5 and Figure 8. Table 5 summarizes the values of the free surface energy (γ_s_), along with its polar (γ_p_) and displacement (γ_d_) components, as calculated using the OWRK method [42,43,44].

After comparing the results, it can be stated that the free surface energy increased as the radiation dose increased, which resulted in the significant improvement in the adhesion properties of the surface layers of the investigated materials. From the free surface energy viewpoint, the most suitable radiation dose for LDPE and HDPE was considered to be 165 kGy, since it led to the highest increase in free surface energy for both HDPE and LDPE. The free surface energy of HDPE increased from 24.4 mJ/m^2^ (for the non-irradiated material) to 42.6 mJ/m^2^ (for the material irradiated using the radiation dose of 165 kGy), which represented an increase of approximately 75%. Irradiation increased the free surface energy of LDPE by approximately 94%, from 23.5 to 45.6 mJ/m^2^. The radiation dose of 66 kGy was the most appropriate for PP, and it increased its free surface energy from 26.3 to 41.4 mJ/m^2^, which represented an increase of approximately 57%. The free surface energy values (see Table 5 and Figure 8) were in good agreement with the measured wetting contact angle values.

The materials used in this study, HDPE, LDPE, and PP, are low-surface-energy polymeric materials [2,4]. From the obtained results, modification by using β^−^ radiation can be considered to be an effective method for achieving high surface energies for the studied polymers. According to the literature [4], the high energy surface of polymeric materials must have energy higher than 40 mJ/m^2^. All tested materials met this condition after being irradiated, using their respective optimal radiation doses (see Table 5).

**Figure 9:**
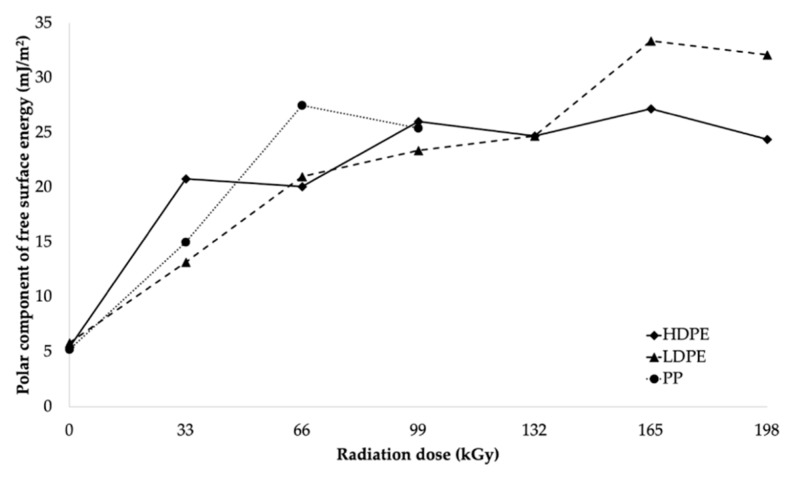
Polar component’s dependence on radiation dose.

In addition to the total free-surface-energy value, its polar component also plays a significant role in the resultant adhesion. The influence of the dose of absorbed radiation on the polar component of the free surface energy of all materials tested is illustrated in Figure 9. β^−^ radiation significantly increased the value of the polar component of the free surface energy. Not unlike the other tested properties, the highest increase in the polar component of the free surface energy for HDPE and LDPE was achieved by using the radiation dose of 165 kGy. The polar component of the free surface energy of HDPE increased from 5.4 to 27.2 mJ/m^2^, which was an increase of approximately 404%. Moreover, irradiation increased the polar component of the free surface energy of LDPE from 5.8 to 33.4 mJ/m^2^, which was an increase of approximately 476%. The most appropriate radiation dose for PP appeared to be 66 kGy. This increased its polar component of the free surface energy from 5.2 to 27.5 mJ/m^2^, which represented an increase of approximately 429%.

In addition to wettability and free surface energy, the presence of reactive functional groups, such as carbonyl and hydroxyl, on the surface of the bonded material can significantly affect the ultimate tensile strength of bonded joints [1,2,5,20]. Infrared spectroscopy was used to determine the contributions of these groups to the ultimate tensile strength of bonded joints.

**Figure 10:**
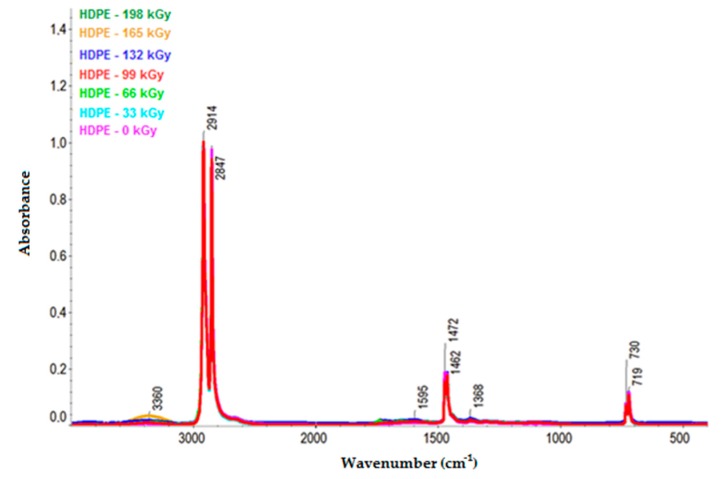
Infrared spectra of HDPE.

**Figure 11:**
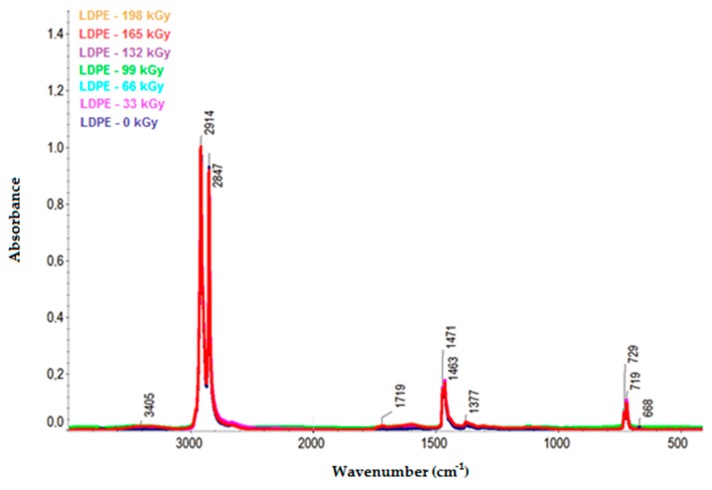
Infrared spectra of LDPE.

Figure 10 and Figure 11 illustrate the infrared spectra of HDPE and LDPE, respectively. The HDPE spectra depict the decrease in intensity of the bands ascribed to aliphatic CH bonds (e.g., the negative bands at 3000–2800, ~1450, and ~1370 cm^−1^) and the increase in intensity of the bands corresponding to oxygenated functional groups at 1595 and 3360 cm^−1^. Furthermore, the LDPE spectra illustrate the increase in bandwidth of the oxygen-functional groups at 1719 and 3405 cm^−1^.

**Figure 12:**
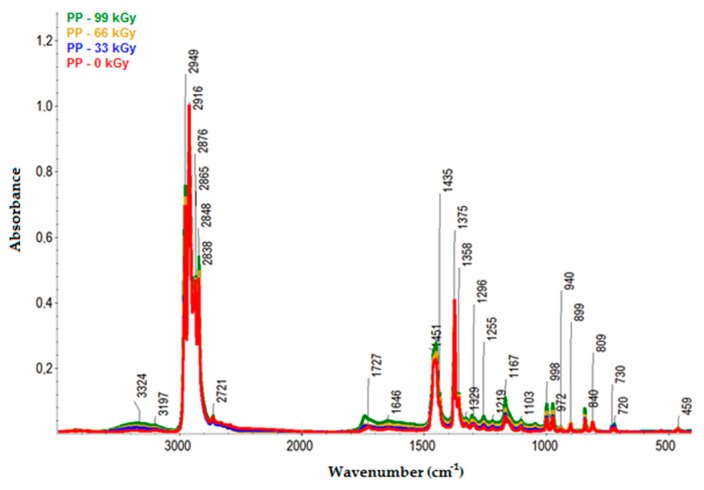
Infrared spectra of PP.

The infrared spectra of PP are presented in Figure 12. Like HDPE and LDPE, since PP was oxidized during irradiation, hydroxyl (3600–3000 cm^−1^ range), and carbonyl (1800–1500 cm^−1^ range) groups were identified in its spectra.

**Figure 13:**
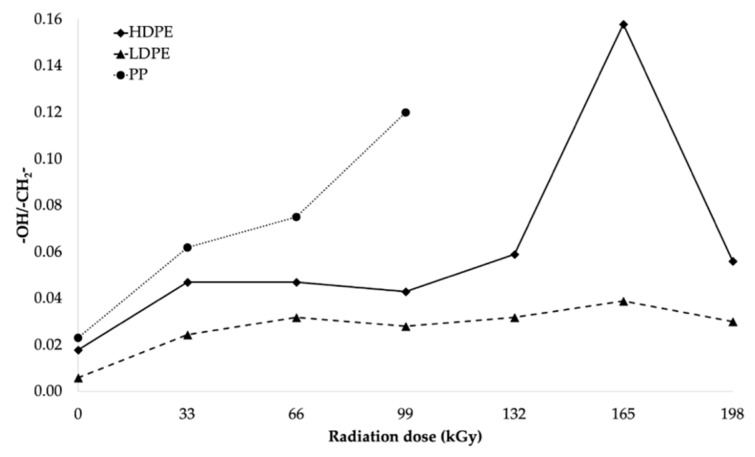
Relationships between relative number of hydroxyl functional groups and radiation dose.

**Figure 14:**
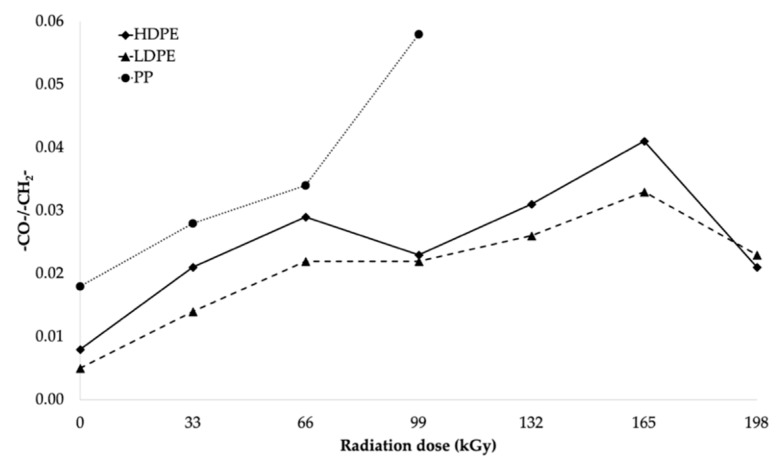
Relationship between relative number of carbonyl-functional groups and radiation dose.

From the infrared spectra, we concluded that irradiation caused the oxidation of CH bonds (–CH_2_–) in HDPE, LDPE, and PP, and carbonyl (–CO–) and hydroxyl (–OH) groups were formed as a result. The increase in the relative numbers of oxygen functional groups is presented in Figure 13 and Figure 14 (for hydroxyl and carbonyl groups, respectively).

From Figure 13 and Figure 14, it was concluded that the relative numbers of hydroxyl and carbonyl groups in all tested materials depended on the dose of absorbed radiation. The lowest relative number of hydroxyl groups was recorded for the non-irradiated materials. The relative number of oxygen-containing groups increased as the radiation dose increased. The highest increase in the relative number of oxygen-containing groups for HDPE and LDPE was observed for the radiation dose of 165 kGy, while the radiation dose that generated the highest increase in the relative number of oxygen-containing groups for PP was 99 kGy (see Figure 13).

The trends in the relative number of carbonyl and hydroxyl groups (see Figure 14 and Figure 13, respectively) were similar. The relative number of carbonyl groups increased as the radiation dose increased. The highest increase in the relative number of oxygen-containing groups for HDPE and LDPE was observed at 165 kGy, while the highest increase for PP was observed at 99 kGy (see Figure 14).

Similar to the changes in wetting contact angles and free surface energy, the changes in the properties of the surface layers of all tested materials were probably due to oxidation which occurred during and after beta irradiation. Oxidation is one of the secondary reactions that occurs when ionizing radiation interacts with a polymer [21], and it results in the formation of the abovementioned carbonyl- and hydroxyl-functional groups. The kinetic processes that cause the degradation of these products are most likely controlled by atmospheric oxygen diffusion throughout the bulk polymer.

Alkyl and allyl radicals reacted with oxygen molecules to form carbonyl groups on the surfaces of samples, while, within the samples, the alkyl radicals led to crosslinking and the allyl radicals reacted with oxygen to form hydroperoxide groups. These findings were in agreement with the results reported by Murray [47] and Hama [48]. In addition to the oxidation, which affects the surface properties of polymers, polymer properties are also influenced by post-irradiation oxidation. Carpentieri [49] and Costa [50] mentioned that post-irradiation oxidation is one of the factors that influences the crystallinity and size of crystalline lamellas of polymers. This could explain, to a certain extent, some of the differences in the relative numbers of oxygenated functional groups of HDPE and LDPE (shown in Figure 13 and Figure 14, respectively).

A lower relative number of oxygenated functional groups was observed for LDPE, which presented lower crystallinity than HDPE. These results could be attributed to the differences in reactivity between macro-alkyl radicals, which could form in the amorphous and crystalline phases of the polymers during irradiation. Rivaton [51] suggested that the macro-alkyl radicals that formed in the amorphous phase decayed very rapidly. Nevertheless, other macro-allyl radicals could migrate from the crystalline phase at the amorphous/crystalline interface, where they would become more accessible to oxygen, which would subsequently lead to the development of post-irradiation oxidation.

Therefore, it can be concluded that, for LDPE, post-irradiation oxidation was very low and was achieved in a relatively short time. The lamellae of LDPE are very thin lamellae compared to those of HDPE, and therefore the migration time of the macro-alkyl radicals from the crystalline to the amorphous phase was very short. Conversely, the oxidation rate of HDPE was higher, and it was reached after a relatively long time after the irradiation process. The wider crystalline lamellae of HDPE captured the macro-allyl radicals, and, thus, their migration time to the amorphous phase was longer. Hence, the migration time of the radicals from the crystalline to the amorphous phase appeared to be one of the key factors governing the oxidation process.

### 3.3. Load-Bearing Capacity of Bonded Joints

Using the measured surface-layer properties, it was concluded that beta irradiating HDPE, LDPE, and PP caused the oxidation of their surface layers, which resulted in improved wettability, increased free surface energy, and increased the relative oxygen content of the surface. Therefore, these changes became the main factors that positively influenced the adhesion properties of the tested materials, which manifested in the extreme increase of the bond strength of the bonded joints.

The maximum load forces that the bonded joints were able to transfer were measured to assess the load-bearing capacity of the bonded joints. The load-bearing capacities of the bonded joints of HDPE, LDPE, and PP at different absorbed radiation doses are summarized in Table 6, Table 7 and Table 8, respectively. The results are presented as arithmetic means ($\bar{x}$), and the corresponding measurement errors are presented using the standard error of the mean ($S_{\bar{x}}$).

From the results for HDPE and LDPE (shown in Table 6 and Table 7, respectively), it was concluded that, from the perspective of the adhesive bonds, higher radiation doses (99–198 kGy) were required for the materials that exhibited the highest increase in load-bearing strength. The maximum adhesive bond strength of HDPE (see Table 6) was observed when Cyberbond 2028 adhesive was used at the radiation dose of 165 kGy. For such a bonded joint, the average value of the maximum load-bearing force increased from 185.1 N (for the non-irradiated material) to 840.6 N (for the material irradiated using the dose of 165 kGy), which represented an increase of approximately 354%. The most suitable combination of Cyberbond 2008 adhesive and 198 kGy radiation dose was obtained for LDPE (see Table 7), but from a technical viewpoint, the optimal radiation dose was 165 kGy. For such a bonded joint, the average maximum load-bearing force increased from 111.8 to 375.6 N, which represented an increase of approximately 236% (at the radiation dose of 198 kGy), and from 111.8 to 372.2 N, which represented an increase of 232% at the lower radiation dose of 165 kGy.

For PP (see Table 8), it cannot be unequivocally stated that higher radiation doses were associated with the highest increase in the average maximum load-bearing force, since extreme increases have also been achieved at the lowest radiation dose. For this type of material, the maximum bond strength of the bonded adhesive was observed by using the combination of Cyberbond 2028 adhesive and radiation dose of 99 kGy, when the average maximum load-bearing force increased from 388.8 to 1001.6 N, which represented an increase of approximately 158%. However, the highest increase in the average maximum load-bearing force of 440% was obtained using the two-component methacrylate adhesive Plexus MA300 and irradiation dose of 66 kGy.

The results of the load-bearing capacity of the bonded joints were in agreement with those of the surface-layer-properties tests. Furthermore, the results were in agreement with the measured gel content, where the gel content of LDPE and HDPE was reported to be minimal at lower radiation doses (see Figure 6), while PP presented an extreme increase in its gel content at the lowest radiation dose (see Figure 6). These results indicated that β^−^ radiation appeared to be a very effective tool for increasing the load-bearing capacity of bonded HDPE, LDPE, and PP joints. 

## 4. Discussion

**Figure 15:**
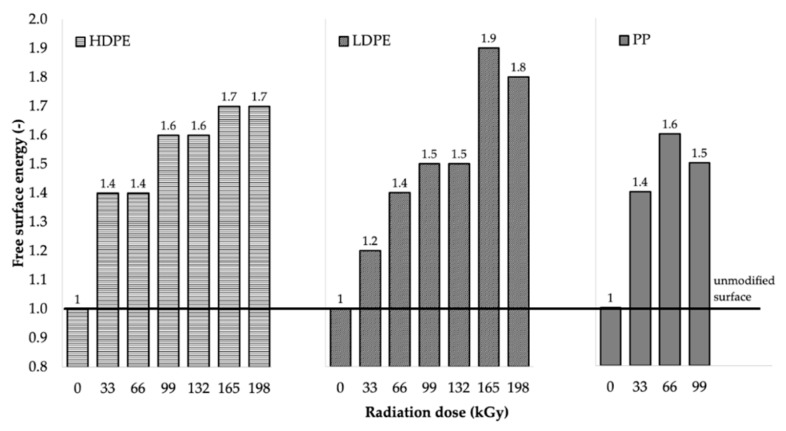
Dependence of free surface energy change on radiation dose.

This study focused on the influence of radiation on surface-layer properties (wetting contact angle, free surface energy, relative number of functional groups, and adhesiveness) of selected types of polymers. Two types of PE, LDPE and HDPE, and one type of PP were selected as test materials. These polyolefins are known for their low surface energy and poor adhesion properties; therefore, they cannot be glued together without prior surface treatment. The results indicated that β^−^ radiation greatly improved wettability, as reported by the decrease in wetting contact angles of the irradiated samples compared to the non-irradiated ones. The decrease in wetting contact angles occurred for all studied materials and used reference liquids. Moreover, it was determined that the free surface energy of these polyolefins increased as the radiation dose increased, which resulted in significant improvements in the adhesive properties of the polymers. The most appropriate radiation dose for increasing the free surface energy of HDPE and LDPE appeared to be 165 kGy, where an increase of up to 90% was recorded, while the increase in the free surface energy of PP was approximately 60% and was observed at the radiation dose of 66 kGy (see Figure 15). A similar trend was observed for the polar component of the free surface energy, which did not contribute significantly to the resulting strength of the glued bonds, either. Increases of at least 400% in the polar component of the free surface energy were observed for all investigated materials, and they were attributed to irradiation. Infrared spectroscopy quoted the effect of β^−^ radiation on increasing the relative numbers of carbonyl and hydroxyl groups. The relative numbers of functional groups increased as the radiation dose increased for all investigated materials. Moreover, the highest relative numbers of functional groups for LDPE and HDPE were determined at the radiation dose of 165 kGy, while, for PP, this occurred at the dose of 66 kGy.

The changes in the surface-layer properties were due to the oxidation that occurred during and after irradiating the samples using β^−^ radiation. As a consequence of these changes, the load-bearing capacities of the bonded joints increased significantly.

**Figure 16:**
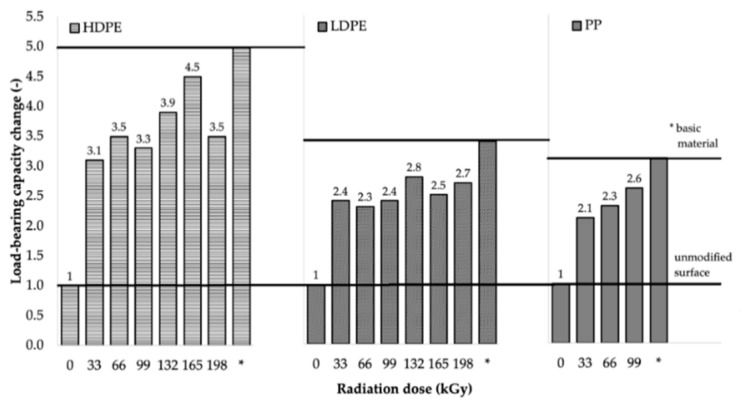
Dependence of bonded-joint load-bearing capacity change on radiation dose (Cyberbond 2028 was used as adhesive).

The Cyberbond 2028 adhesive appeared to be the most appropriate adhesive of all, since samples bound using this adhesive exhibited the largest changes in load-bearing capacity (see Figure 16). 

**Figure 17:**
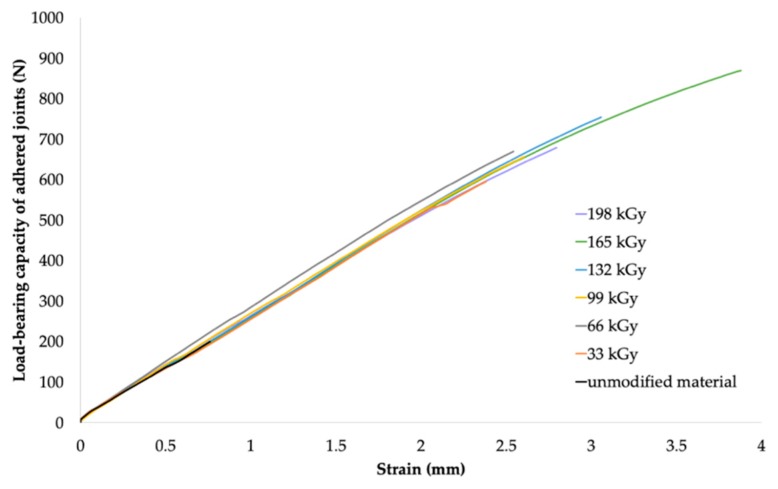
Dependence of bonded-joint load-bearing capacity of adhered joints on strain (HDPE and Cyberbond 2028).

The bond strength of the HDPE sample irradiated using the radiation dose of 165 kGy was approximately 90% of the strength of the original polymer. As a result of the surface modification of the bonded materials, the bond strength of the adhesive bonds of the irradiated HDPE samples increased by up to 4.5 times compared to the non-irradiated HDPE sample (see Figure 16 and Figure 17). 

**Figure 18:**
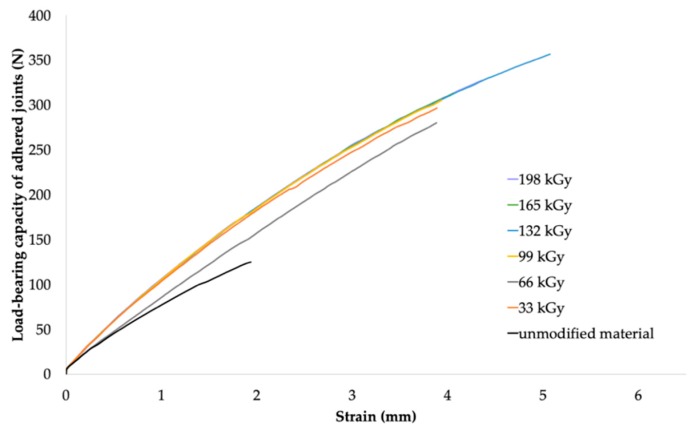
Dependence of bonded-joint load-bearing capacity of adhered joints on strain (LDPE and Cyberbond 2028).

Very good results were also obtained for the adhered LDPE joints. The bond strength of the LDPE sample bonded using the same adhesive (Cyberbond 2028) and dose of radiation (132 kGy) was 80% of the strength of the original polymer (see Figure 16 and Figure 18).

**Figure 19:**
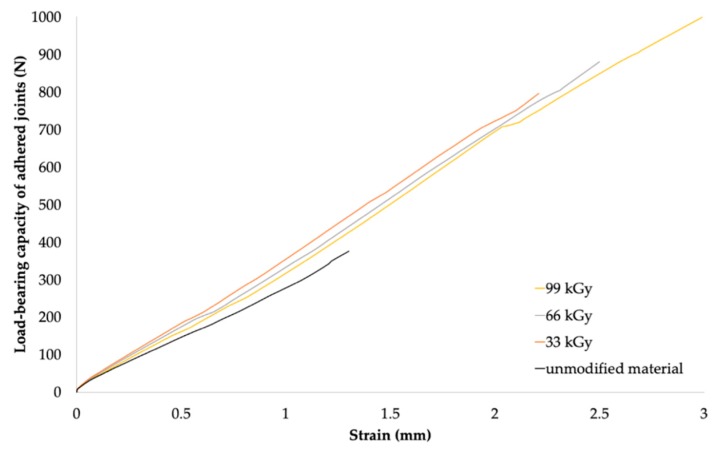
Dependence of bonded-joint load-bearing capacity of adhered joints on strain (PP and Cyberbond 2028).

The resistance of PP irradiated using the radiation dose of 99 kGy was also high; it was up to 85% of the strength of the original polymer (see Figure 16 and Figure 19). In contrast, adhered bonds which used two-component adhesives, such as methacrylate and epoxy-type adhesives, exhibited much lower load-bearing capacities compared to those formed using the cyanoacrylate adhesive (see Table 8).

## 5. Conclusions

The tests carried out in this study allowed us to conclude the following:

- Irradiation favorably affected the wettability and surface energy of the tested polymers.
- Β^−^ radiation significantly improved the adhesive properties of the tested polymers.
- Modification of surface properties using β^−^ radiation appeared to be a very effective tool for improving of bond strength of HDPE-, LDPE-, and PP-bonded polymer parts.

From the application viewpoint, it is necessary to individually determine the most suitable dose of radiation with respect to the requirements of the bonded joint, carefully select the most suitable type of adhesive, and consider the economy of the entire process.

## Figures and Tables

**Table 1:** Tested polymers.

Polymer Type	Trade Name	Company
LDPE	DOW LDPE 780 E	DOW (Midland, MI, USA)
HDPE	DOW HDPE 25055 E	DOW (Midland, MI, USA)
PP	PP V-PTS-CREALEN-EP2300L1*M800	PTS (Adelshofen, Germany)

**Table 2:** List and designation of adhesives used.

Adhesive Group	Adhesive Manufacturer	Adhesive Designation
Cyanoacrylate adhesives	Cyberbond	1008
	Cyberbond	2008
	Cyberbond	2028
	Cyberbond	5008
Two-component methacrylate adhesives	Plexus	MA 300
	Plexus	MA 832
	Cyberbond	A 806
Two-component epoxide adhesive	Cyberbond	E 705

**Table 3:** Injection molding parameters.

Processing Conditions	LDPE	HDPE	PP
Injection velocity (mm/s)	50	60	50
Injection pressure (MPa)	60	80	80
Injection time (s)	0.4	0.4	0.5
Holding pressure (MPa)	50	60	40
Holding time (s)	30	30	10
Cooling time (s)	30	20	40
Mould temperature (°C)	40	40	50
**Plastic Unit Temperature Bands**			
Zone 1 (°C)	190	200	210
Zone 2 (°C)	200	205	220
Zone 3 (°C)	210	210	230
Zone 4 (°C)	215	225	240

**Table 4:** Wetting contact angles of HDPE, LDPE, and PP materials (depending on radiation dose).

Material	Reference Liquid	Radiation Dose (kGy)						
		0	33	66	99	132	165	198
**HDPE**	Distilled Water	(89.2 ± 0.3)°	(67.8 ± 0.4)°	(69.4 ± 0.3)°	(63.1 ± 0.4)°	(61.6 ± 0.5)°	(58.0 ± 0.4)°	(60.0 ± 0.4)°
	Glycerine	(79.1 ± 0.3)°	(64.0 ± 0.4)°	(64.3 ± 0.4)°	(60.4 ± 0.4)°	(58.7 ± 0.5)°	(54.6 ± 0.4)°	(54.4 ± 0.4)°
	Ethylene Glycol	(66.3 ± 0.4)°	(46.4 ± 0.5)°	(49.8 ± 0.4)°	(43.0 ± 0.5)°	(36.2 ± 0.4)°	(31.1 ± 0.5)°	(33.1 ± 0.4)°
**LDPE**	Distilled Water	(89.2 ± 0.3)°	(78.5 ± 0.3)°	(71.4 ± 0.6)°	(68.2 ± 0.4)°	(66.4 ± 0.4)°	(54.7 ± 0.5)°	(57.6 ± 0.5)°
	Glycerine	(79.2 ± 0.5)°	(71.5 ± 0.5)°	(67.3 ± 0.6)°	(62.7 ± 0.3)°	(62.0 ± 0.5)°	(49.3 ± 0.3)°	(53.1 ± 0.5)°
	Ethylene Glycol	(67.9 ± 0.3)°	(59.3 ± 0.5)°	(55.6 ± 0.4)°	(53.5 ± 0.4)°	(50.5 ± 0.5)°	(36.6 ± 0.5)°	(41.3 ± 0.4)°
**PP**	Distilled Water	(88.1 ± 0.5)°	(68.9 ± 0.4)°	(59.5 ± 0.4)°	(60.9 ± 0.4)°			
	Glycerine	(76.7 ± 0.4)°	(58.4 ± 0.4)°	(55.9 ± 0.5)°	(56.5 ± 0.4)°			
	Ethylene Glycol	(63.9 ± 0.5)°	(41.7 ± 0.5)°	(36.6 ± 0.4)°	(37.2 ± 0.5)°			

**Table 5:** Free surface energy and its elements for HDPE, LDPE, and PP materials (depending on radiation dose).

Material	Free Surface Energy and Its Elements (mJ/m^2^)	Radiation Dose (kGy)						
		0	33	66	99	132	165	198
**HDPE**	γ_s_	24.4	34.8	33.7	38.3	39.7	42.6	41.4
	γ_s_^p^	5.4	20.8	20.1	26.0	24.7	27.2	24.4
	γ_s_^d^	19.0	14.0	13.6	12.3	15.0	15.4	17.0
**LDPE**	γ_s_	23.5	27.7	31.8	34.4	35.8	45.6	43.2
	γ_s_^p^	5.8	13.2	21.0	23.4	24.7	33.4	32.1
	γ_s_^d^	17.7	14.5	10.8	11.0	11.1	12.2	11.1
**PP**	γ_s_	26.3	37.0	41.4	40.3			
	γ_s_^p^	5.2	15.0	27.5	25.4			
	γ_s_^d^	21.1	22.0	13.9	14.9			

**Table 6:** Load-bearing capacity of adhered joints for HDPE material (depending on radiation dose and adhesive).

Adhesive		Radiation Dose (kGy)						
		0	33	66	99	132	165	198
Cyberbond 1008	$\bar{x}\;\left(N\right)$	117.2	342.3	388.1	378.9	447.3	571.1	513.9
	$s_{\;\bar{x}\;}\left(N\right)$	3.6	14.8	25.0	14.2	19.3	19.7	23.2
Cyberbond 2008	$\bar{x}\;\left(N\right)$	153.7	287.9	307.3	366.2	361.3	502.2	394.0
	$S_{\;\bar{x}\;}\left(N\right)$	8.0	17.3	13.9	28.8	21.7	19.2	10.3
Cyberbond 2028	$\bar{x}\;\left(N\right)$	185.1	582.6	649.2	616.3	717.0	840.6	653.9
	$S_{\;\bar{x}\;}\left(N\right)$	16.7	33.7	21.5	40.9	35.1	15.2	28.8
Cyberbond 5008	$\bar{x}\;\left(N\right)$	109.3	244.6	234.4	314.4	343.8	382.6	409.2
	$S_{\;\bar{x}\;}\left(N\right)$	6.3	16.2	13.1	12.3	22.1	14.1	31.2
Cyberbond A806	$\bar{x}\;\left(N\right)$	105.4	191.2	209.4	221.8	252.0	235.4	225.5
	$S_{\;\bar{x}\;}\left(N\right)$	5.1	9.7	11.9	10.1	17.1	9.6	13.2
Plexus MA300	$\bar{x}\;\left(N\right)$	136.1	286.4	270.6	474.7	538.4	522.5	550.0
	$S_{\;\bar{x}\;}\left(N\right)$	18.0	19.0	34.3	25.4	13.8	32.5	20.8
Plexus MA832	$\bar{x}\;\left(N\right)$	57.3	140.1	179.9	170.5	200.3	251.1	199.8
	$S_{\;\bar{x}\;}\left(N\right)$	1.4	8.0	3.6	4.2	9.5	8.0	11.1
Cyberbond E705	$\bar{x}\;\left(N\right)$	124.8	189.4	233.7	301.8	240.9	299.1	246.5
	$S_{\;\bar{x}\;}\left(N\right)$	3.5	5.4	12.8	12.0	7.7	7.6	15.7

**Table 7:** Load-bearing capacity of adhered joints for LDPE material (depending on radiation dose and adhesive).

Adhesive		Radiation Dose (kGy)						
		0	33	66	99	132	165	198
Cyberbond 1008	$\bar{x}\;\left(N\right)$	114.3	233.1	250.7	242.2	256.2	330.6	307.0
	$S_{\;\bar{x}\;}\left(N\right)$	6.7	10.0	8.1	9.5	12.3	10.9	12.5
Cyberbond 2008	$\bar{x}\;\left(N\right)$	111.8	168.0	220.1	320.0	322.0	372.2	375.6
	$S_{\;\bar{x}\;}\left(N\right)$	4.7	4.6	13.7	14.7	15.0	19.4	14.2
Cyberbond 2028	$\bar{x}\;\left(N\right)$	119.8	282.6	271.7	290.6	337.0	302.8	322.6
	$S_{\;\bar{x}\;}\left(N\right)$	11.0	15.2	7.3	14.6	16.2	10.5	8.0
Cyberbond 5008	$\bar{x}\;\left(N\right)$	81.2	138.9	173.0	277.8	261.2	258.1	270.8
	$S_{\;\bar{x}\;}\left(N\right)$	2.7	9.2	11.9	8.1	12.2	10.9	14.1
Cyberbond A806	$\bar{x}\;\left(N\right)$	78.7	112.1	152.2	147.2	207.3	184.6	179.3
	$S_{\;\bar{x}\;}\left(N\right)$	3.6	4.3	6.0	5.7	10.0	9.7	4.1
Plexus MA300	$\bar{x}\;\left(N\right)$	64.4	119.6	129.8	168.6	176.8	179.4	225.9
	$S_{\;\bar{x}\;}\left(N\right)$	8.8	8.3	18.4	14.6	6.1	15.1	10.9
Plexus MA832	$\bar{x}\;\left(N\right)$	49.5	88.2	101.2	137.5	131.0	180.8	204.7
	$S_{\;\bar{x}\;}\left(N\right)$	5.1	1.4	4.4	6.0	1.1	8.0	8.7
Cyberbond E705	$\bar{x}\;\left(N\right)$	112.0	154.3	173.3	173.9	251.0	264.5	253.1
	$S_{\;\bar{x}\;}\left(N\right)$	3.8	5.5	7.5	11.8	9.2	15.8	10.1

**Table 8:** Load-bearing capacity of adhered joints for PP material (depending on radiation dose and adhesive).

Adhesive		Radiation Dose (kGy)			
		0	33	66	99
Cyberbond 1008	$\bar{x}\;\left(N\right)$	195.1	352.0	494.7	450.5
	$S_{\;\bar{x}\;}\left(N\right)$	7.1	21.4	16.6	16.7
Cyberbond 2008	$\bar{x}\;\left(N\right)$	374.7	910.8	857.4	810.5
	$S_{\;\bar{x}\;}\left(N\right)$	20.2	32.1	26.4	38.1
Cyberbond 2028	$\bar{x}\;\left(N\right)$	388.8	800.4	905.1	1001.6
	$S_{\;\bar{x}\;}\left(N\right)$	15.8	35.9	28.2	41.3
Cyberbond 5008	$\bar{x}\;\left(N\right)$	119.9	199.9	182.0	152.6
	$S_{\;\bar{x}\;}\left(N\right)$	3.5	7.1	6.6	3.2
Cyberbond A806	$\bar{x}\;\left(N\right)$	112.5	108.6	160.9	194.6
	$S_{\;\bar{x}\;}\left(N\right)$	5.4	6.7	6.9	8.1
Plexus MA300	$\bar{x}\;\left(N\right)$	132.7	686.5	724.9	699.6
	$S_{\;\bar{x}\;}\left(N\right)$	5.7	27.9	30.3	23.0
Plexus MA832	$\bar{x}\;\left(N\right)$	73.2	198.9	213.0	199.4
	$S_{\;\bar{x}\;}\left(N\right)$	2.6	7.0	11.7	8.8
Cyberbond E705	$\bar{x}\;\left(N\right)$	177.0	292.3	401.8	610.1
	$S_{\;\bar{x}\;}\left(N\right)$	3.7	15.8	22.7	20.6
